# NF-κB in T-cell Acute Lymphoblastic Leukemia: Oncogenic Functions in Leukemic and in Microenvironmental Cells

**DOI:** 10.3390/cancers2041838

**Published:** 2010-11-05

**Authors:** Nuno R. dos Santos, Marinella N. Ghezzo, Ricardo C. da Silva, Mónica T. Fernandes

**Affiliations:** IBB-Institute for Biotechnology and Bioengineering, Centre for Molecular and Structural Biomedicine (CBME), University of Algarve, Campus de Gambelas, 8005-139 Faro, Portugal

**Keywords:** NF-κB, signaling, T cell, leukemia, lymphoma, NOTCH1, microenvironment

## Abstract

Two main NF-κB signaling pathways, canonical and noncanonical, performing distinct functions in organisms have been characterized. Identification of mutations in genes encoding components of these NF-κB signaling pathways in lymphoid malignancies confirmed their key role in leukemogenesis. T-cell acute lymphoblastic leukemia (T-ALL) is an aggressive malignancy of thymocytes that despite significant therapeutic advances can still be fatal. Although mutations in NF-κB genes have not been reported in T-ALL, NF-κB constitutive activation in human T-ALL and in acute T-cell leukemia mouse models has been observed. Although these studies revealed activation of members of both canonical and noncanonical NF-κB pathways in acute T-cell leukemia, only inhibition of canonical NF-κB signaling was shown to impair leukemic T cell growth. Besides playing an important pro-oncogenic role in leukemic T cells, NF-κB signaling also appears to modulate T-cell leukemogenesis through its action in microenvironmental stromal cells. This article reviews recent data on the role of these transcription factors in T-ALL and pinpoints further research crucial to determine the value of NF-κB inhibition as a means to treat T-ALL.

## 1. Introduction

Initially identified as regulators of immunoglobulin genes, the NF-κB proteins have been found to be involved in multiple physiological and cellular processes. In addition, NF-κB deregulation has been linked with a wide spectrum of human pathologies [1]. Germline or somatic alterations in human genes encoding either NF-κB proteins or upstream regulators have been identified in cancer, inflammatory conditions, immunodeficiencies, and skin and bone malformation syndromes. Several studies have uncovered key roles for NF-κB proteins in solid and hematological malignancies, turning this signaling pathway into a potential therapeutic target [2,3]. Recent findings have indicated that this is also true for T-cell acute lymphoblastic leukemia (T-ALL), as discussed in the present review. Although initially identified as activated in T-ALL cells, this signaling pathway can also contribute to leukemogenesis through its function in microenvironmental cells, as mentioned below.

## 2. Canonical and Noncanonical NF-κB Activation Pathways

The NF-κB family comprises five members (RelA, RelB, c-Rel, NF-κB1/p50, and NF-κB2/p52) that combine as homo- or heterodimers to bind DNA and regulate transcription [4,5]. All family members contain the characteristic Rel homology domain (RHD), responsible for DNA binding, dimerization, and nuclear localization. The RelA (p65), RelB, and c-Rel subunits contain transactivation domains (TADs) that interact with transcriptional coactivators to control gene expression. The p50 and p52 proteins, which derive from proteolytic processing of the p105 and p100 precursor proteins, respectively, do not contain transactivation domains, and can only control gene transcription through dimerization with other NF-κB subunits or interaction with other transcriptional regulators (e.g., Bcl3). In the steady-state the NF-κB dimers are localized in the cytoplasm bound to inhibitory IκB proteins. The IκB proteins (IκBα, IκBβ, IκBε, p100, p105, and Bcl3) are characterized by the presence of an ankyrin repeat domain, which interacts with and inhibits the RHD domain of NF-κB proteins. Thus, only when IκB proteins are degraded or proteolytically processed upon cell stimulation and IκB kinase (IKK) activation, do NF-κB factors translocate to the nucleus and become activated.

**Figure 1:**
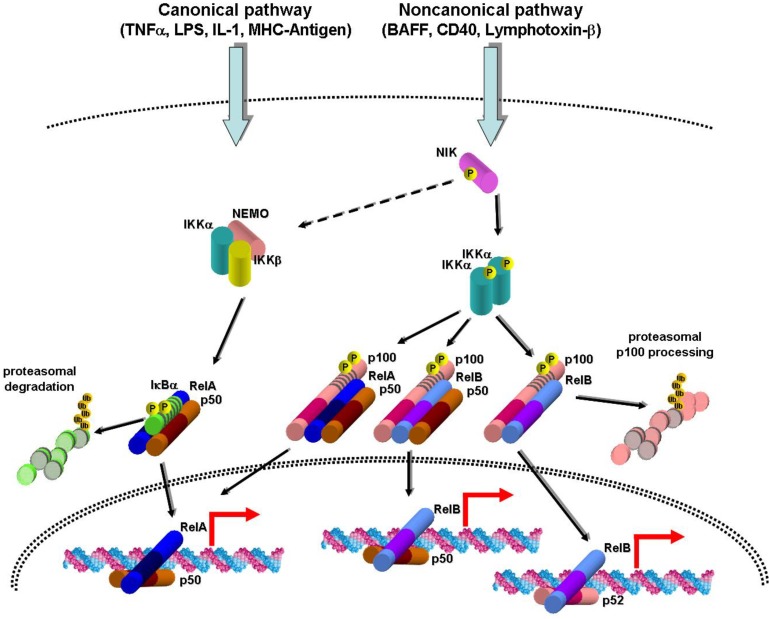
Schematic representation of the main actors intervening in the canonical and noncanonical NF-κB activation pathways. Stimuli such as TNFα, lipopolysaccharide (LPS), interleukin-1 (IL-1), and major histocompatibility complex (MHC)-coupled antigen activate, through intermediary proteins, an IKK complex including the α and β catalytic subunits and the NEMO regulatory subunit. Activation of this complex entails phosphorylation of IκBα thus promoting its subsequent ubiquitination and proteasomal degradation. The nuclear localization signal present in the p50:RelA heterodimer becomes exposed, allowing its translocation to the nucleus and interaction with DNA target elements. The noncanonical pathway is activated by TNF family members such as BAFF, CD40, and lymphotoxin-α_1_β_2_ and stabilizes and activates the NIK kinase through inhibition of intermediary proteins including TRAF2 and TRAF3. The NIK kinase has the ability to activate not only the noncanonical IKKα/IKKα complex but also the canonical IKK complex [26]. The IKKα complex phosphorylates p100 on C-terminal serines resulting in proteasomal degradation of p100 bound to p50:RelA and p50:RelB dimers or proteasomal processing of p100 bound to p52. This leads to nuclear translocation of p50:RelA, p50:RelB, and p52:RelB heterodimers, and regulation of their target genes.

Two main NF-κB pathways have been described that are activated by distinct stimuli, trigger distinctive transcriptional programs, and participate in diverse biological functions (Figure 1) [4,5]. The canonical pathway relies on the activation of the NEMO/IKKα/IKKβ kinase complex by tumor necrosis factor (TNF)α and other proinflammatory cytokines, Toll-like receptors, antigen receptors, DNA damage, among other signals, and is involved in biological processes such as cell survival, stress response, and inflammation. The IKK complex phosphorylates IκBα, IκBβ, or IκBε on serine residues and earmarks these proteins for ubiquitination and proteasomal degradation. This pathway leads to activation of mainly RelA- or c-Rel-containing dimers. Despite the presence of IKKα in IKK complexes, gene inactivation studies have shown that only the NEMO regulatory subunit (also known as IKKγ) and the IKKβ catalytic subunit are required for canonical NF-κB activation [6,7,8,9,10,11]. Upon activation, RelA and c-Rel undergo serine phosphorylation at their TADs, allowing interaction with the CBP/p300 transcriptional co-activators, and transcriptional activation. Termination of canonical NF-κB signaling occurs through different mechanisms acting at different levels (reviewed by Vallabhapurapu and Karin [5]). These include IκBα re-synthesis with consequent export of RelA-containing dimers to the nucleus, ubiquitin-mediated RelA proteasomal degradation triggered by its phosphorylation by IKKα and implemented by SOCS1 and COMMD1 or PDLIM2 proteins, RelA displacement from DNA by PIAS proteins, and inactivation of the IKK complex or its upstream regulators by the A20 and CYLD deubiquitinating enzymes.

The noncanonical NF-κB pathway, which is important for lymphoid organogenesis, B-cell maturation, and bone development, relies on the activation of the NF-κB-inducing kinase (NIK) and IKKα kinases by a subset of TNFα-related receptors (e.g., BAFF, CD40, and LTβR). NIK is constitutively degraded by the proteasome through TRAF3-mediated ubiquitination by cIAP proteins. Upon receptor activation, TRAF3 and the associated TRAF2, cIAP1, and cIAP2 proteins are degraded, and NIK is stabilized [12,13,14]. Then NIK mediates recruitment of IKKα to p100 [15], and the latter is phosphorylated on serine residues and subsequently ubiquitinated and proteolytically processed to the p52 subunit. It was recently demonstrated that p100 functions (through its ankyrin repeat domain) as an inhibitor of several NF-κB dimers, including p50:RelA and p50:RelB [16,17]. Therefore, when p100 undergoes proteolysis, not only RelB- but also RelA-containing dimers are activated, although with slow kinetics, as compared to RelA heterodimers activated through the canonical pathway [17,18]. Studies using genetically deficient cells have indicated that NIK and IKKα, but not NEMO and IKKβ, are directly required for noncanonical NF-κB activation [16,18,19,20]. However, since expression of the RelB and p100 genes is induced by canonical NF-κB [21,22], inactivation of this pathway also hampers noncanonical signaling [23]. As recently unraveled, noncanonical signaling can be terminated through the IKKα-mediated phosphorylation of COOH-terminal serines of NIK that promote its destabilization, through a TRAF3-independent mechanism [24].

The noncanonical activation of p50:RelA dimers by TNF-related receptors appears to occur through NIK/IKKα-mediated p100 degradation (Figure 1) [17]. NIK may, however, activate RelA through other mechanisms. In lymphoid cells, NIK was shown to be required for IκBα phosphorylation induced by the TNF family receptors CD27, CD40, and BAFF-R, but not by TNFα itself [25]. Activation of canonical NF-κB by TNF-related receptors appears to involve NIK-mediated recruitment of the IKKα/IKKβ/NEMO complex to these receptors and its subsequent activation [25]. Another study showed that increased IκBα phosphorylation in *Traf3*-deficient mouse embryonic fibroblasts was dependent on NIK protein stabilization [26]. Although these data pointing to NIK activation by canonical NF-κB signaling may not reflect physiological settings, they are relevant in pathological situations when NIK is constitutively stabilized (see below).

## 3. NF-κB Activation in Leukemia and Lymphoma

Both canonical and noncanonical NF-κB activation through different mechanisms has been reported in several types of human hematological malignancies, chiefly lymphoid leukemia and lymphoma (reviewed by Jost and Ruland [27] and Packham [28]). Constitutive NF-κB activation often results from rearrangements and mutations in NF-κB genes or in genes encoding upstream components of the signaling pathway. Initial reports described chromosomal deletions or translocations leading to *NFKB2* gene rearrangements in cutaneous T-cell lymphoma, B-cell non-Hodgkin lymphoma, chronic lymphocytic leukemia, and multiple myeloma [27,28]. More recently, genetic alterations in components of the noncanonical and canonical NF-κB pathways have been identified in a significant number of multiple myeloma cases [29,30]. Indeed, gain-of-function alterations were found in the *NFKB1*, *NFKB2*, *NIK,CD40*, *LTBR*, and *TACI* genes. In other cases, loss-of-function mutations were found in the *TRAF2*, *TRAF3*, *CYLD*, and *BIRC2/BIRC3* genes, which encode negative regulators of NF-κB. Several of these mutations were found in genes encoding regulators of the noncanonical NF-κB pathway, including NIK, the NIK-activating CD40, TACI, and LTβR receptors, and members of the complex that interacts with NIK and triggers its proteasomal degradation (*i.e.*, TRAF2, TRAF3, BIRC2/cIAP1, and BIRC3/cIAP2) [13,14]. However, not only noncanonical but also canonical NF-κB activity was found to be increased in multiple myeloma samples [29]. Consistently, IKKα and IKKβ inhibitory studies showed that IKKβ-mediated canonical NF-κB signaling is important for multiple myeloma cell proliferation and survival [29,31]. Mutations in genes encoding NF-κB regulators were also found in the activated B-cell-like subtype of diffuse large B-cell lymphomas, including mutations inactivating the NF-κB negative regulator *A20/TNFAIP3* and mutations activating the *CARD11*, *TRAF2*, *TRAF5*, *TAK1*, and *RANK* positive regulators of NF-κB [32,33,34].

NF-κB activation in leukemia/lymphoma may also derive from other mechanisms such as persistent autocrine or paracrine signaling. For example, ligand-independent signaling from overexpressed CD30 [35], CD40 stimulation by paracrine (T cell-derived) CD40L stimulation [36], or autocrine RANK, BAFF, or APRIL stimulation [37,38,39]. Oncogenic kinase activity can also activate NF-κB in leukemia, as demonstrated for BCR-ABL [40,41,42] and TEL-PDGFRβ fusion proteins [43]. Finally, proteins from viral strains associated with hematological malignancies (e.g., Epstein-Barr virus and human T-lymphotropic virus type 1) have the ability to activate canonical and noncanonical NF-κB pathways [27,28].

## 4. Molecular Pathogenesis of T-cell Acute Lymphoblastic Leukemia

T-cell acute lymphoblastic leukemia (T-ALL) and T-cell lymphoblastic lymphoma (T-LBL) are aggressive malignancies of thymocytes that affect mainly children and adolescents. Although clinically distinct, T-ALL and T-LBL are often grouped together due to their similar morphological, genetic, and immunophenotypic features [44,45], and therefore will be referred to here simply as T-ALL. Being a thymocyte neoplastic disease, T-ALL seemingly originates in the thymus, at least in some cases. T-ALL patients frequently present high peripheral blast counts, central nervous system dissemination and larger mediastinal masses that cause tracheal compression and respiratory distress at diagnosis. Fortunately, current chemotherapeutic regimens can cure most pediatric and many adult patients, albeit with substantial secondary effects.

Several recurrent genetic alterations have been identified in human T-ALL [46,47,48,49]. Chromosomal translocations occur in about 20% of cases and result either in fusions between the coding regions of two genes, leading to chimeric protein expression, or in fusions between proto-oncogenes and T-cell receptor (TCR) loci, leading to oncogene overexpression (e.g., *TAL1*, *TLX1*, *TLX3*, *HOXA*, *LMO1*, and *LMO2*). One of the most frequent alterations in T-ALL (more than 50% of cases) are *NOTCH1* mutations, leading to activation of NOTCH1-dependent transcriptional programs [50]. Deletion or inactivating mutations in the *CDKN2A* gene occur in about 70% of cases, and these lead to loss or haploinsufficiency of its encoding proteins, the p16^INK4a^ and ARF tumor suppressor proteins [51]. Although less frequently, other genetic alterations have been detected in T-ALL, including activating mutations in genes encoding the JAK1 [52], N-RAS [53], and FLT3 [54] signaling proteins, *NUP214*-*ABL1* gene fusions [55], *MYB* gene duplications [56,57], inactivating mutations in *FBW7* (which encodes an ubiquitin ligase that triggers degradation of NOTCH1 among other proteins) [58], inactivating mutations and deletions in *PTEN* [59,60], *LEF1* inactivation [61], *PTPN2* deletions [62], and *PHF6* mutations [63]. Activation of several signaling pathways, including PI3K/Akt, MAPK, JAK-STAT, and NF-κB has also been reported in T-ALL (reviewed by Cardoso *et al.* [64] and Staal and Langerak [65]).

## 5. NF-κB Activation in Human T-cell Acute Lymphoblastic Leukemia and T-cell Leukemia/Lymphoma Mouse Models

Although mutations in NF-κB genes have not been reported in T-ALL, unlike other lymphoid malignancies, NF-κB constitutive activation can occur in human T-ALL and mouse models of acute T-cell leukemia. NF-κB constitutive activation was initially detected by Kordes and colleagues [66], who detected NF-κB activity in human T-ALL primary samples (11 of 13 cases) by electrophoretic mobility shift assays. Antibody supershift analysis identified p50:p50 homodimers and p50:RelA heterodimers activated in these cells. The T-ALL primary samples also presented phosphorylated IκBα, indicative of canonical IKK activity. More recently, when analyzing a series of human T-ALL cell lines, Vilimas *et al*. [67] also found constitutive NF-κB DNA-binding activity. These investigators also detected constitutive IKK kinase activity and nuclear localization of the p50, p105, RelA, RelB, and c-Rel subunits in T-ALL cell lines. These data, together with findings that NF-κB is constitutively activated in transgenic mouse models of T-ALL induced by Notch1, Notch3, Tal1, and TEL-JAK2 oncoproteins [68,69,70,71], indicate that NF-κB activation occurs frequently in T-ALL leukemic cells. Furthermore, the reported RelB nuclear localization in T-ALL cell lines [67] suggests that not only canonical but also noncanonical NF-κB activation can occur in T-ALL. However, this notion remains to be confirmed since increased p100 processing, the hallmark for noncanonical NF-κB activation, is yet to be reported for this malignancy.

## 6. NF-κB Inhibition in Human and Murine Leukemic T Cells

To evaluate the impact of NF-κB activity on the leukemic phenotype of T-ALL, Vilimas *et al.* [67] treated human T-ALL cell lines with NF-κB canonical pathway inhibitors. Most cell lines treated with either BMS-345541, an IKKβ inhibitor, or bortezomib, a proteasome inhibitor, underwent apoptosis [67]. Furthermore, specific blockade of the canonical IKK complex with the NEMO-binding domain cell-permeable peptide inhibited NF-κB activity and led to apoptosis of T-ALL cell lines [71]. Apoptosis also occurred when NF-κB was inhibited by IκBα overexpression in a leukemic T-cell line derived from transgenic Notch3 mouse lymphoma [68]. In addition, murine leukemic T-cell lines resistant to chemotherapeutic agents and displaying constitutive NF-κB activation underwent apoptosis upon treatment with BAY11-7086, an inhibitor of IκBα phosphorylation [72]. Together, these studies indicate that canonical IKK/NF-κB signaling is essential for T-ALL cell viability. However, it remains to be verified whether primary patient samples are similarly sensitive to NF-κB inhibition and whether noncanonical NF-κB signaling also plays a specific role in T-ALL cell survival or proliferation.

Although the impact of NF-κB inhibition on human T-ALL growth in xenograft mouse models is yet to be investigated, T-cell leukemogenesis in mice engrafted with syngenic bone marrow progenitors transduced with intracellular NOTCH1 protein (ICN1) was shown to be impaired by either T cell-specific expression of an undegradable IκBα mutant (IκBαΔN) protein [67] or hematopoietic lineage deletion of NEMO [71]. Based on blood cell counts and histological analyses, Vilimas *et al.* [67] concluded that impaired leukemogenesis by IκBαΔN was due to reduced infiltration of several tissues by leukemic cells. The effects of NEMO deletion in the hematopoietic lineage on ICN1-mediated leukemogenesis appeared more drastic than those caused by IκBαΔN overexpression in the T-cell lineage. Upon NEMO deletion, mice having received Mx1-Cre-positive bone marrow progenitors retrovirally transduced with ICN1 presented reduced leukemic blasts in the blood, reduced spleen and liver infiltration, and increased leukemic cell apoptosis than NEMO-proficient controls [71]. These experiments unequivocally demonstrated that canonical IKK kinase activity is essential for Notch1-induced mouse T-ALL. However, since Mx1-Cre-mediated NEMO deletion inactivates NF-κB in all hematopoietic cells, the possibility remains that NF-κB inactivation in cells other than leukemic T cells may contribute to leukemogenesis, similarly to the role of inflammatory cells in carcinoma mouse models [73]. It would thus be interesting to verify whether NF-κB activation in hematopoietic microenvironmental cells also contributes to T-ALL.

NF-κB inhibition in mice has, however, not always resulted in T-ALL regression. Indeed, despite being associated with constitutive NF-κB activation, leukemogenesis driven by the TEL-JAK2 fusion protein was not prevented or weakened by expression of serine-mutated undegradable IκBα protein in the murine T-cell lineage [69]. Likewise, IκBα mutant expression in leukemic T cells derived from TAL1 transgenic mice, which present increased p50:RelA DNA-binding activity and NEMO-associated kinase activity, did not prevent tumor formation in syngenic recipient mice [70]. IκBα mutant protein was functional in leukemic T cells from both TAL1 and TEL-JAK2 transgenic mice, because its expression effectively blocked NF-κB activation induced by TNFα or phorbol ester/ionomycin [69,70]. These studies showing that NF-κB inhibition mediated by mutant IκBα protein failed to impair T-cell leukemogenesis in two mouse models, hints that at least some subtypes of acute T-cell leukemia may not require canonical NF-κB activation for its development or maintenance. However, one cannot exclude the possibility that NF-κB activation in these mouse models either was not completely abolished by mutant IκBα or it does not depend on IκBα degradation, depending rather on an alternative mechanism of activation. Consistent with this notion, several studies indicate that NF-κB activity can be regulated by post-translational modifications of NF-κB or by interactions with other proteins [74].

## 7. Paracrine/Autocrine Mechanisms of NF-κB Activation in T-ALL

**Figure 2:**
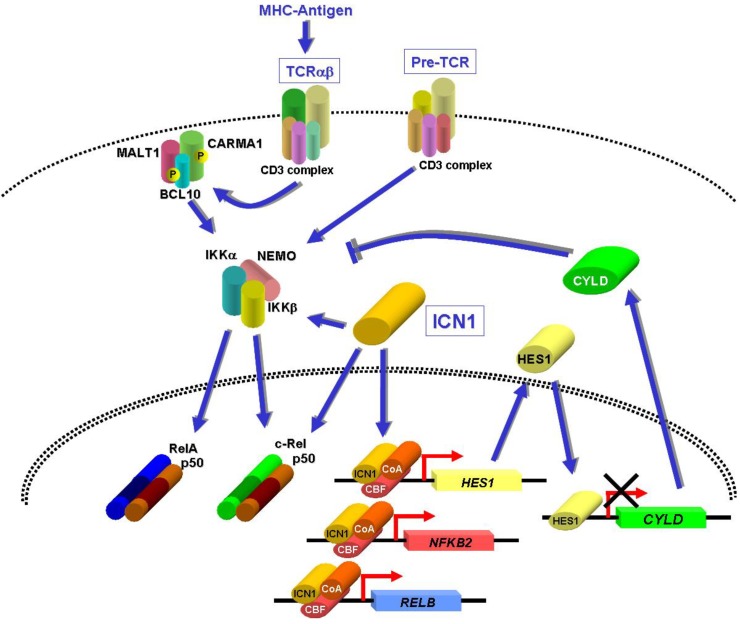
Potential mechanisms of NF-κB activation in T-ALL leukemic cells. TCRαβ and pre-TCR cell surface complexes are known to activate NF-κB in T cells and may do so in T-ALL as well, through constitutive signaling (pre-TCR) or MHC-coupled antigenic stimulation (TCRαβ). Intracellular NOTCH1 protein (ICN1) was shown to interact with and enhance IKK activity in T-ALL cells [67] and to interact with and stabilize p50:c-Rel heterodimers in the nucleus of activated splenocytes [89]. The ICN1-induced HES1 repressor facilitates NF-κB activation by repressing the expression of the deubiquitinating enzyme CYLD [71], a negative regulator of the canonical NF-κB pathway. Together with CBF and co-activators (CoA), ICN1 may potentially facilitate noncanonical NF-κB activation by direct induction of the *NFKB2* and *RELB* genes [67,88].

The mechanisms of NF-κB and IKK activation in T-ALL cells are beginning to be unveiled. In parallel to other lymphoid malignancies, these mechanisms may be intrinsic to neoplastic cells, due to mutations affecting the intracellular components of the NF-κB pathway, as discussed in the following sections, or may depend on incoming microenvironmental cues. In normal T cells NF-κB is activated by TCR or pre-T cell receptor (pre-TCR) signaling [75,76,77]. TCR stimulation by antigen results in oligomerization of the CARMA1, BCL10, and MALT1 (CBM) complex, which activates the IKK complex [5,78]. Although MALT1 and CARMA1 genetic alterations activating these proteins and activating NF-κB have been found in B-cell lymphomas [32,34,79,80], no mutations affecting the CBM complex in T-ALL have so far been reported. Since TCR overstimulation can be oncogenic [81], TCR expression could potentially favor leukemogenesis through NF-κB activation, paralleling the recently discovered role of B-cell receptor-mediated NF-κB activation in diffuse large B-cell lymphoma [82]. Although cell surface TCR expression appears to be infrequent in T-ALL samples and cell lines, about half of primary cases express cytoplasmic TCR chains [83,84]. In addition, several T-ALL cases express the pTα protein [83], which together with the TCRβ chain is an essential component of the pre-TCR complex. It is thus possible that TCR or pre-TCR expression drives NF-κB activation in human T-ALL (Figure 2), although supporting evidence, at least in human cells, is lacking. TCRα deficiency in TEL-JAK2 transgenic mice led to a reduction in RelA DNA-binding activity in leukemic T cells [69]. However, this decrease in RelA activity was not associated with a delay in leukemia onset, suggesting that RelA was not essential for TEL-JAK2-induced disease [69]. Another study showed that pre-TCR expression was important for canonical NF-κB activation in leukemic T cells from transgenic Notch3 mice [85]. Supporting the notion that pre-TCR may drive NF-κB activity in T-ALL, Vacca *et al.* [85] further showed that pTα deficiency attenuated the expression of NF-κB target genes putatively linked to the leukemic phenotype. In sum, these findings obtained with mouse models support the hypothesis that pre-TCR rather than TCR expression contributes to the activation of NF-κB in leukemic T cells.

## 8. NF-κB Activation by NOTCH1 in T-ALL

The NF-κB signaling pathway can be potentially activated by oncoproteins expressed in T-ALL leukemic cells. *NOTCH1* mutations found in human T-ALL result in increased levels of intracellular NOTCH1 protein and activation of NOTCH1-dependent transcriptional programs [86,50]. Several reports have indicated that NOTCH1 can activate NF-κB at different levels (reviewed by Osipo *et al*. [87]) (Figure 2). Through its DNA-binding interaction partner CBF1/RBP-Jκ, NOTCH1 was shown to bind the promoter and induce the expression of *NFKB2* and *RELB* in T-ALL cells [67,88]. In addition, ICN1 was shown to interact directly with and to retain p50 and c-Rel in the nucleus of activated splenocytes, thus preventing their sequestration by IκBα and resulting in sustained NF-κB activity [89]. Finally, NOTCH1 was shown to interact with and enhance IKK activity in T-ALL and cervical carcinoma cells [67,90]. Another mechanism for ICN1-induced IKK/NF-κB activation was recently uncovered by Espinosa *et al.* [71], who found that induction of the Hes1 transcriptional repressor by ICN1 in T-ALL cells led to CYLD downregulation and concomitant increased IKK activity. Endogenous Hes1 protein could bind the *CYLD* promoter in T-ALL cells, and the CYLD expression levels were found to be significantly decreased in most human primary T-ALL samples analyzed. This contrasted to *HES1* expression, which was increased in these cells. The experiments performed by Espinosa and collaborators [71] in T-ALL and HEK293T cells support the notion that HES1-induced CYLD repression results in increased IKK kinase activity, IκBα degradation, RelA nuclear translocation, and NF-κB transcriptional activity. No mutations in the *CYLD* gene were identified in T-ALL patients, indicating that in contrast to the multiple myeloma setting, where *CYLD* mutations were detected [29,30], this gene is inhibited at the transcriptional level. Finally, the finding that NOTCH1 inhibition in T-ALL cell lines by γ-secretase inhibitors (GSI) reduced the activity of both IKK [67] and an NF-κB-dependent reporter [71] demonstrated the causative relationship between *NOTCH1* mutations and IKK/NF-κB activity.

## 9. NF-κB Activation by Other Oncoproteins?

However important ICN1 may be for NF-κB activation, it is possibly not the sole means of activating these transcription factors in T-ALL. Genome-wide gene expression studies have led to the definition of molecular subgroups of T-ALL, characterized by the ectopic expression of particular proto-oncogenes (e.g., *TAL1*, *LMO1*, *LMO2*, *TLX1*, *TLX3*, and *HOXA*) and associated different disease prognosis (extensively reviewed by van Vlierbergh *et al.* [49]). Although no NF-κB pathway gene signature was detected for the TAL1-positive subgroup, other work showed that TAL1 protein expression in T-ALL cells can control NF-κB activity through direct *NFKB1* promoter repression [91]. The lower steady-state p50 levels in T-ALL cell lines caused by TAL1 expression impaired p50:RelA dimer formation and increased c-Rel:RelA dimers when cells were exposed to etoposide, an NF-κB-activating agent [91]. Etoposide-induced ICAM1 expression, a target of c-Rel:RelA heterodimers, was eliminated by antisense-mediated TAL1 knock-down, supporting the notion that transcriptional programs may be modulated by different types of NF-κB dimers present in T-ALL cells. Whether different NF-κB dimers are activated in different molecular subgroups of T-ALL should be investigated.

NF-κB transcription factors may be activated by other signaling pathways. Constitutive JAK2 kinase activity due to fusion with the TEL/ETV6 transcriptional repressor has been linked with human and murine B-cell and T-cell malignant transformation [92,93,94,95,96]. By an as yet undefined mechanism, TEL-JAK2 kinase activity was shown to activate IKK proteins and to induce NF-κB DNA-binding activity in the Ba/F3 B-cell line [97,98]. Although leukemic T cells from TEL-JAK2 transgenic mice presented NF-κB DNA-binding activity [69] no evidence for a direct activation of NF-κB by this fusion protein in human or mouse leukemic T cells has so far been reported.

The possibility that NF-κB activation in T-ALL is caused by other genetic alterations found in this disease warrants investigation, especially concerning proteins known to regulate NF-κB in other settings. For example the *LCK* gene has been found to be overexpressed due to rare chromosomal translocations involving the *TCRB* locus in T-ALL [99] and its kinase activity can activate NF-κB in T cells [100]. Increased ABL1 kinase activity occurs in a subset of T-ALL patients due to the *NUP214-ABL1* gene fusion [55], and another ABL1 fusion, BCR-ABL1, has been shown to activate NF-κB in myeloid leukemic cells [101,102]. Finally, PI3K/Akt pathway activation due to PTEN inactivation occurs in many T-ALL cases [59,60] and this pathway has also been shown to activate NF-κB in T lymphocytes [103]. Alterations in the MAP kinase ERK5 gene have not yet been reported in human T-ALL, but the ERK5 protein has been shown to be important for leukemic T cell survival and ability to grow in immunodeficient mice [104]. In addition, ERK5 was able to induce RelA nuclear localization and to activate an NF-κB reporter gene, through an uncharacterized mechanism independent of canonical IKK activation [104]. In sum, several molecular alterations may concurrently or individually increase NF-κB activity. Since no specific mutations affecting directly the NF-κB pathway have been detected in T-ALL, NF-κB is most likely activated by a combination of factors.

## 10. NF-κB Function in Microenvironmental Cells of T-ALL

Accumulating evidence indicates that cancer progression depends not only on alterations in cancer cells but also on microenvironmental factors [105]. Heterotypic signaling can occur in hematological malignancies [106], yet little is known about the microenvironmental factors participating in T-ALL. NF-κB activation in microenvironmental cells has been pinpointed as a key player in the genesis of a variety of cancers [73], and recent reports indicate that NF-κB activity in microenvironmental cells can also contribute to T-ALL pathogenesis. It was recently found that RelB deficiency in non-hematopoietic stromal cells impaired murine leukemogenesis driven by the TEL-JAK2 fusion protein [69]. Since T-cell leukemogenesis in the transgenic TEL-JAK2 mouse model, like human T-ALL, appears to originate from thymocytes [92,107], RelB-dependent thymic stromal cells are the most likely non-hematopoietic cells involved in this disease. RelB-deficient mice present subtle defects in the thymic microenvironment, such as absence of a defined medulla and absence of medullary thymic epithelial cells (mTEC) [108,109,110]. Compared to controls, RelB-deficient mice also present a strong reduction in CD80^+^DEC205^+^dendritic cell (DC) numbers, which was shown to be secondary to the defects in thymic architecture and mTECs [111]. Despite these thymic defects, thymocyte development up to the CD4^+^CD8^+^ double-positive stage appeared unimpaired in RelB-deficient mice (combined with TCRα deficiency), indicating that the potential targets for TEL-JAK2-induced malignant transformation were not lacking [69]. These results have thus pinpointed a role for RelB in T-cell leukemogenesis through its activity in microenvironmental cells, presumably localized in the thymus.

**Figure 3:**
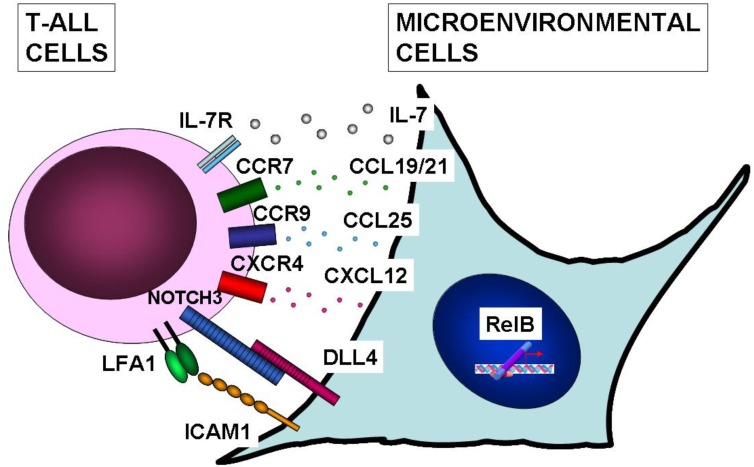
Microenvironmental signals assisting T-ALL leukemic cells. IL-7 produced by stromal cells was shown to induce survival and proliferation of T-ALL cells [114,115]. T-ALL cells were shown to express cognate receptors and to respond to CCL19/CCL21, CCL25 and CXCL12 chemokines, which are potentially induced by RelB [18]. CCR7 stimulation by CCL19 or CCL21 was shown to direct T-ALL cells to the mouse central nervous system [113]. CCR9 stimulation by CCL25 induced T-ALL chemotaxis and resistance to chemotherapy-induced apoptosis [127], while CXCR4 stimulation by CXCL12 also induced T-ALL chemotaxis [128]. Another study showed that the NOTCH3 ligand, Dll4, mediated T-ALL escape from tumor dormancy in mice [116]. Stromal cells expressing the ICAM1 adhesion molecule were shown to favor *in vitro* survival of T-ALL cells expressing LFA-1 integrin [129]. Whether the expression and/or function of these or other stromally produced proteins is controlled by RelB or other NF-κB subunits remains to be determined.

The mechanisms through which RelB supports T-cell leukemogenesis remain blurred (Figure 3). CCR7 stimulation is a likely candidate, since its ligands CCL19 and CCL21 are induced by RelB-dependent noncanonical NF-κB signaling [112]. CCR7 expression was recently reported in human primary T-ALL and cell lines [113], and animal studies showed that CCR7 stimulation by its ligands was crucial for the targeting and infiltration of leukemic T cells to the central nervous system [113]. Regarding other potential mechanisms, no evidence has been raised indicating that RelB or other NF-κB members induce NOTCH ligands or IL-7, which are important thymic microenvironmental and oncogenic factors in T-ALL [114,115,116,117]. Further investigation is thus warranted to identify the RelB-dependent microenvironmental molecular cues important for T-ALL development.

## 11. NF-κB Inhibition as a Therapeutic Strategy for T-ALL?

T-ALL patients are treated by multiagent chemotherapy protocols, that include glucocorticoids (e.g., prednisone or dexamethasone), anthracyclins (e.g., doxorubicin or daunorubicin), the alkaloid vincristine, asparaginase, alkylating agents (e.g., cyclophosphamide), and antimetabolites (e.g., nelarabine, cytarabine, mercaptopurine and methotrexate) [118]. Treatment with these compounds results in leukemic cell apoptosis [119]. In addition, several of these agents (e.g., vincristine, daunorubicin, doxorubicin, and cytarabine) are known to induce NF-κB activation, due to their DNA damaging action [120,121]. NF-κB activation in different cancers has been shown to confer resistance to chemotherapy-induced apoptosis [121]. Supporting the notion that constitutive NF-κB activity indeed protects cancer cells from chemotherapy-induced apoptosis, several studies demonstrated that NF-κB inhibition through expression of the IκBα super-repressor mutant or pharmacological NF-κB inhibitors rendered chemoresistant cancer cell lines sensitive to chemotherapeutic agent-induced apoptosis [121]. Moreover, anti-apoptotic NF-κB target genes, such as pro-survival Bcl2 family members and the IAP family of caspase inhibitors, were found to be expressed in chemoresistant cancer cell lines with constitutive NF-κB activation [121,122].

NF-κB activation may also confer chemoresistance to T-ALL cells. When Garcia *et al.* [72] compared murine leukemic T-cell lines resistant to vincristine or doxorubicin with the chemosensitive parental cell line, they found that the former presented higher constitutive NF-κB activity. This suggested that higher NF-κB activity could underlie chemoresistance in these cells. BAY11-7082-mediated NF-κB inhibition led to apoptosis of chemoresistant cell lines and abrogated resistance to vincristine or doxorubicin, indicating that this pathway is crucial for chemoresistance [72]. Another study showed that DNA damage induced by doxorubicin, etoposide, and other agents activated NF-κB in the CEM T-ALL cell line [123]. These investigators also showed that NF-κB blockade by the IκBα super-repressor led to CEM apoptosis upon DNA damage induction, indicating that chemoresistance in this cell line depends on its ability to activate NF-κB [123].

Although needing confirmation with primary samples, these reports indicate that NF-κB inhibitors (e.g., proteasomal or IKKβ inhibitors) can induce T-ALL apoptosis and can be of therapeutic value either as stand-alone therapy or as an additional tool to improve standard chemotherapy regimens currently in use. NF-κB inhibitors may also be useful in combination with inhibitors of other signaling pathways activated in T-ALL, such as the NOTCH1 and mTOR pathways [124,125,126]. However, therapy using NF-κB/IKKβ inhibitors can only be envisaged when firm pre-clinical or clinical evidence that the canonical NF-κB activation is important for the maintenance of human T-ALL *in vivo* is obtained.

## 12. Conclusions

As reviewed here, accumulating evidence obtained with different experimental models points to a role for NF-κB signaling in T-ALL. Importantly, the pro-oncogenic roles of NF-κB appear to be both intrinsic to leukemic T cells and mediated by microenvironmental cells. Several issues remain however to be elucidated. Different studies have provided evidence for both canonical and noncanonical NF-κB activation in T-ALL cells, but it has not been clearly determined how these pathways impinge on the malignant features of T-ALL *in vivo*. This is not a minor issue since these pathways differ in terms of regulation, kinetics, and activated target genes. Furthermore, NF-κB inhibitors currently being tested in clinical trials target the canonical pathway and may thus not be effective to block noncanonical NF-κB activity in T-ALL. Experiments aiming to inhibit either canonical or noncanonical NF-κB signaling in animal models of human T-ALL using genetically inactivated alleles or RNA interference are warranted to address this issue. Such experiments should also shed light on the nature of NF-κB target genes that contribute to the malignant phenotype.

How NF-κB activity in the T-ALL microenvironment participates in this disease is still unclear. It will be important to determine whether NF-κB or RelB activity in microenvironmental cells is important throughout different stages of disease. If so, one can envisage T-ALL therapy through NF-κB inhibition in leukemia-associated microenvironmental cells. Normal microenvironmental cells are likely less prone to mutate and develop resistance to therapy than malignant cells.
